# A negative association between horn length and survival in a weakly dimorphic ungulate

**DOI:** 10.1002/ece3.6050

**Published:** 2020-02-26

**Authors:** Mathieu Douhard, Jean‐Paul Crampe, Anne Loison, Christophe Bonenfant

**Affiliations:** ^1^ Laboratoire d’Écologie Alpine UMR CNRS 5553 Université Savoie Mont‐Blanc Le Bourget‐du‐Lac France; ^2^ Parc National des Pyrénées Tarbes France; ^3^ Laboratoire de Biométrie et Biologie Évolutive UMR CNRS 5558 Université Lyon 1 Villeurbanne France

**Keywords:** horn growth, life‐history tactics, longevity, *Rupicapra*, sexual selection, survival, weapon

## Abstract

While all models of sexual selection assume that the development and expression of enlarged secondary sexual traits are costly, males with larger ornaments or weapons generally show greater survival or longevity. These studies have mostly been performed in species with high sexual size dimorphism, subject to intense sexual selection. Here, we examined the relationships between horn growth and several survival metrics in the weakly dimorphic Pyrenean chamois (*Rupicapra pyrenaica*). In this unhunted population living at high density, males and females were able to grow long horns without any apparent costs in terms of longevity. However, we found a negative relationship between horn growth and survival during prime age in males. This association reduces the potential evolutionary consequences of trophy hunting in male chamois. We also found that females with long horns tended to have lower survival at old ages. Our results illustrate the contrasting conclusions that may be drawn when different survival metrics are used in analyses. The ability to detect trade‐off between the expression of male secondary sexual traits and survival may depend more on environmental conditions experienced by the population than on the strength of sexual selection.

## Graphical abstract

In this study, we examined correlations between horn growth and several survival metrics in an unhunted population of Pyrenean chamois. Males and females were able to grow long horns without any apparent costs in terms of longevity. However, males with long horns showed a lower survival during early adulthood than those with smaller horns.

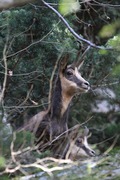

## 1 INTRODUCTION

The heart of life‐history theory is the “principle of allocation,” which states that organisms allocate limited energy either to growth, reproduction, or maintenance (Cody, 1966; Williams, 1966). As the amount of energy allocated to one of these functions reduces allocation to others, trade‐offs should occur (Roff & Fairbairn, 2007; Stearns, 1992). Trade‐offs may manifest themselves in the short‐term between time *t* and *t* + 1 (Bleu, Gamelon, & Sæther, 2016; Hamel et al., 2010), or in the long‐term after a certain amount of physiological damage has accumulated (Kroeger, Blumstein, Armitage, Reid, & Martin, 2018; Moyes et al., 2006). In particular, high allocation to reproduction or growth early in life should reduce the energy available for somatic maintenance, causing a reduction in later‐life survival (Kirkwood & Rose, 1991; Lemaître et al., 2015).

Adult survival is a major determinant of fitness in long‐lived iteroparous species (Gaillard, Festa‐Bianchet, Yoccoz, Loison, & Toïgo, 2000). It is well known that adult females live longer than males in most populations of mammals (Austad & Fischer, 2016). One of the explanations for such difference involves sexual selection (Maklakov & Lummaa, 2013). Sexual selection arises from differences in reproductive success caused by competition for access to mates (Andersson, 1994). Due to anisogamy, sexual selection typically acts more strongly on males than on females. Females often compete more intensely for resources necessary for successful reproduction than for access to mating partners (Clutton‐Brock, 2009). All models of sexual selection assume that secondary sexual traits are costly to produce and maintain (Møller, 1996). Males are more likely to show higher rates of mortality due to development and maintenance of secondary sexual traits and risk of injuries during fights to gain access to estrous females (Maklakov & Lummaa, 2013). In support of this hypothesis, interspecific comparisons (Clutton‐Brock & Isvaran, 2007; Promislow, 1992) showed that sex differences in adult longevity are smaller and less consistent in monogamous species than in polygynous ones, in which male–male competition is more intense. However, population‐level studies reporting sex differences in survival costs of reproduction or growth are scarce and mainly limited to primates including humans (Bolund, Lummaa, Smith, Hanson, & Maklakov, 2016; Hoffman et al., 2008; Penn & Smith, 2007). Moreover, such sex differences are more likely to reflect sex biases in parental care than the intensity of intrasexual competition.

Horns in bovids and antlers in cervids represent one of the most spectacular examples of secondary sexual traits among vertebrates (Darwin, 1871). Males carrying large horns or antlers generally have higher reproductive success (Coltman, Festa‐Bianchet, Jorgenson, & Strobeck, 2002; Kruuk et al., 2002; Robinson, Pilkington, Clutton‐Brock, Pemberton, & Kruuk, 2006; Willisch, Biebach, Marreros, Ryser‐Degiorgis, & Neuhaus, 2015; Newbolt et al., 2017, but see Mainguy, Côté, Festa‐Bianchet, & Coltman, 2009). The reproductive benefits of large horns and antlers derive from advantages during male–male combats (Lincoln, 1994). In addition, these weapons may provide an honest signal of male quality for choosy females (Ezenwa & Jolles, 2008; Vanpé et al., 2007). While antler possession by females is restricted only to Rangifer (reindeer and caribou), horns are present in females of about two‐third of bovid species. Female horns may have evolved through natural selection by conferring an advantage in predator defense or resource competition (Stankowich & Caro, 2009). Alternatively, horns in females could be nonadaptive and persist due to common genetic machinery in both sexes (Lande, 1980). Sexual selection favoring increased trait size in males can result in a correlated response in females that reduces female fitness (Bonduriansky & Chenoweth, 2009). There was evidence of sexually antagonistic selection on horn size in Soay sheep *Ovis aries* (Robinson et al., 2006) but not bighorn sheep *Ovis canadensis* (Poissant, Wilson, Festa‐Bianchet, Hogg, & Coltman, 2008).

Empirical evidence for a negative relationship between horn growth and natural survival is scarce (see Table 1 in Lemaître et al., 2018). Previous studies have mainly focused on species with a high level of sexual dimorphism (e.g., Soay sheep, Robinson et al., 2006; bighorn sheep, Bonenfant, Pelletier, Garel, & Bergeron, 2009; Alpine ibex *Capra ibex*, Bergeron, Festa‐Bianchet, Hardenberg, & Bassano, 2008; Toïgo, Gaillard, & Loison, 2013). Few studies have investigated similar relationships in species with low sexual size dimorphism, possibly because limited horn length is unlikely to impose major energetic costs. However, recent studies in the weakly dimorphic Alpine chamois (*Rupicapra rupicapra*) showed nonsignificant trends toward decreased longevity with increasing horn growth in males and females (Bleu, Loison, & Toïgo, 2014; Corlatti, Storch, Filli, & Anderwald, 2017). Such relationships may be stronger in populations experiencing poor environmental conditions, as reported for survival costs of reproduction (Cohen, Coste, Li, Bourg, & Pavard, 2019).

Here, we investigate the relationships between horn growth and survival in a wild population of Pyrenean chamois (*Rupicapra pyrenaica*) living at persistent high density. The Pyrenean chamois is a long‐lived (up to 21 years of age, Gonzalez & Crampe, 2001), medium‐sized (about 25 kg, Pépin, Faivre, & Menaut, 1996), mountain‐dwelling ungulate that closely resembles Alpine chamois by many aspects of anatomy, behavior, and life history. Data on paternity adequate to estimate the opportunity for sexual selection are lacking in chamois (Corlatti et al., 2015), but the limited sexual size dimorphism (Rughetti & Festa‐Bianchet, 2011b) and absence of sex differences in adult survival (Loison, Festa‐bianchet, Gaillard, Jorgenson, & Jullien, 1999) suggest a lower opportunity for sexual selection than in highly polygynous ungulates such as red deer (*Cervus elaphus*) and bighorn sheep (Vanpé et al., 2008).

After describing horn growth in both sexes, we examined sex‐specific relationships between horn growth during the first 5 years of life and later survival. To do this, we considered three survival metrics: the probability of reaching 9 years of age (metric for medium‐term survival), the probability of reaching 15 years of age (conditional on having survived to age 9; metric for long‐term survival), and longevity. We also tested for an effect of early horn growth (measured as horn growth in the first 2 years of life), relative late horn growth (see methods for further details), and their interaction on survival.

## 2 MATERIALS AND METHODS

### 2.1 The study population and data collection

The study was carried out in the Cauterets valley within the Pyrenees National Park, France (42°45′–42°55′N, 0°05′‐0°15′W). This area stretches over 10,200 ha between 890 and 3,298 m. The climate is oceanic alpine, and most winter precipitation falls as snow above 1,300 m. There are neither large predators nor hunting. The population density was high, reaching 10 animals/km^2^ in summer and 80 animals/km^2^ in winter on the main winter area, leading to a stable population living close to carrying capacity (Crampe et al., 2007). As a result, age at first reproduction was delayed and reproductive success was lower at any age compared to another population of Pyrenean chamois (Bazès) living at lower density (Crampe et al., 2006). The population was mainly monitored by one person (J‐P.C) who searched for skulls of dead animals from 1987 to 2015. Some skulls were also occasionally found by other park rangers. Most of the skulls (87%) were collected during winter in a core area of 1,400 ha encompassing most of the winter grounds of the population. Each year, this core area was searched once or twice a week along fixed transects, from November to April. Winter home ranges are shared by males and females. Thus, we assumed that the probability of finding skulls was equal for the two sexes (Gonzalez & Crampe, 2001). Outside winter, when chamois substantially increase their range, dead animals were sought over the entire area in a less systematic way, for example, by observing the activity of predators and scavengers. Because the skulls of animals aged ≤2 years old are smaller, lighter, and less sturdy than those of adults, they are more likely to be destroyed or carried off by predators and scavengers (Gonzalez & Crampe, 2001). In the survival analyses, we only used horns of individuals that lived more than 4.5 years. These horns are large enough to assume that the size bias for finding horns is nonexistent (for a similar argument, see Loehr, Carey, Hoefs, Suhonen, & Ylönen, 2007). We therefore consider that a random sample of all horns, independently of their length, is present in the collection.

**Figure 1:**
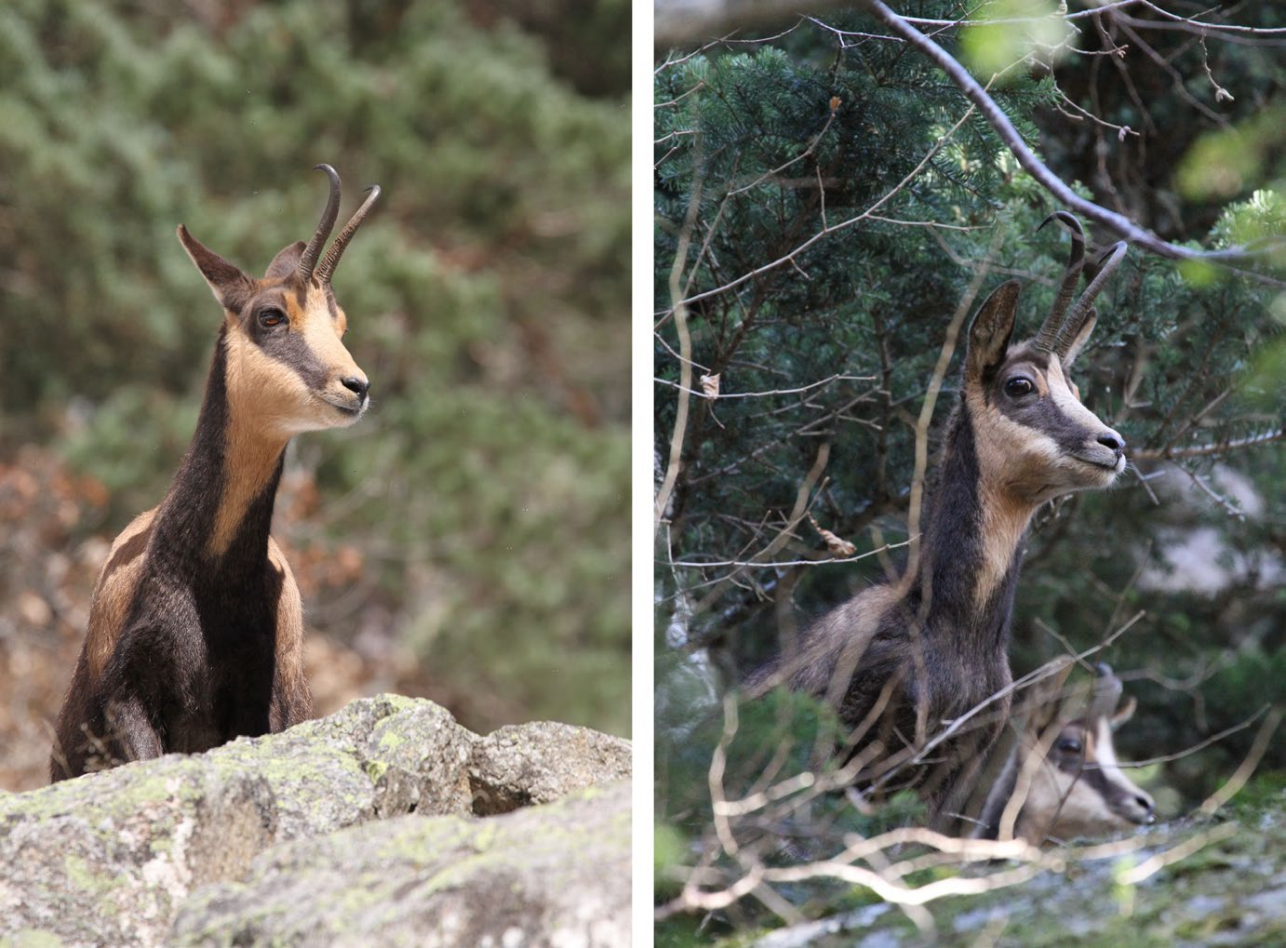
Pyrenean chamois in the study area (left: female; right: male). Photography credit: J‐P.C

For every skull recovered, the same person (J.‐P.C) took several measures. In temperate areas, horns stop growing in winter leading to a pattern of rings called annuli. Longevity can be accurately determined by counting the annuli (Schröder & Elsner‐Schak, 1985). Annual horn increments were measured on the outside curve of the right horn using a flexible measuring tape. Length was taken for first five increments because horn growth reduces to a few millimeters per year after 4.5 years of age. The measure of horn length we have used throughout the paper thus corresponds to the sum of first five increments (growth between birth and 4.5 years). Broken horns were not measured and, in this species with hook‐shaped horns (Figure 1), horn tip wear is considered to be negligible, although it would be informative to quantify wear using repeated measures of horn length on the same individuals at different ages. Horn morphology allowed distinguishing the sexes. The horn base is thicker and the apical hook more pronounced in males than in females (Blagojević & Milošević‐Zlatanović, 2014; Gonzalez & Crampe, 2001). When death was recent, the sex was confirmed from the genitalia.

### 2.2 Statistical analyses

We first ran linear mixed models using the “nlme” library (Pinheiro, Bates, DebRoy, Sarkar, & R Core Team, 2013) in R v. 3.5.1 (http://www.r-project.org) to explore the variation in increment length as a function of age (factor), sex, and their interaction. Individual identity was fitted as a random effect to account for nonindependence of increments from the same individual. We used all individuals in the larger data set for which those data were available (1,760 measures for 253 males and 208 females). Because it is difficult to distinguish horn growth in the first and second year of life in chamois, we combined the first two increments (L1‐L2, see Corlatti et al., 2017; Rughetti & Festa‐Bianchet, 2011a for a similar procedure). To test whether absolute late horn growth was associated with early horn growth, we fitted a linear model for each sex with log‐transformed sum of increments 3, 4, and 5 (L3‐L5) as a function of log‐transformed L1‐L2. We also examined whether log‐transformed horn length was correlated with log‐transformed L1‐L2.

**Figure 2:**
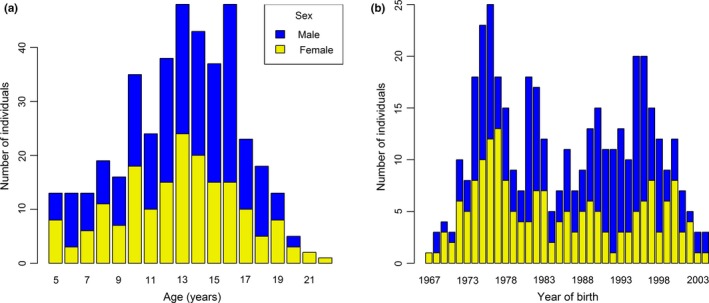
Details of sample sizes for analyses of survival. The number of male and female Pyrenean chamois per (a) age class and (b) per cohort is shown

To assess the relationships between horn length and survival, we calculated three metrics: longevity, probability of reaching 9 years of age, and probability of reaching 15 years of age. This last metric was conditional, that is, it measures the probability that individuals will survive to age 15 given that they have already survived to age 9. We chose 9 and 15 years of age as thresholds because they correspond to first and third quartile of longevity respectively, allowing us to assess medium‐ and long‐term survival impacts. In addition, 9 years is the age at which female survival starts to decrease in this species (Loison et al., 1999). Survival metrics were analyzed with generalized linear mixed models (GLMMs). Longevity was fitted with a Poisson distribution as there was no evidence of overdispersion or underdispersion in data. We modeled the probabilities of reaching 9 and 15 years with a binomial distribution. Each model included fixed effects of sex, horn length, and their interaction, as well as the random effect of cohort. These models estimate values of the sex‐specific effects of horn length, as well as sex differences in these relationships. Longevity and horn length were available for 409 individuals aged ≥5 years (228 males and 181 females). Details of sample sizes are in Figure 2.

To test for survival costs of age‐specific horn growth, we used GLMMs to model each survival metric described above as a function of early horn growth (L1‐L2), relative late horn growth (see definition below), and their interaction. We conducted separate analyses for males and females to avoid the difficulty of providing a biological interpretation of a three‐way interaction. We defined relative late horn growth as the residuals of the regression between log‐transformed L1‐L2 and log‐transformed L3‐L5: A negative value means that individuals grow more slowly than average beyond 1.5 years of age, while a positive value means that individuals experience a higher than average growth beyond 1.5 years of age.

We reported standardized regression coefficients (β) obtained by centering and scaling (mean = 0, variance = 1) all continuous explanatory variables in order to assess effect sizes. Centering enables also interpretation of the main effects involved in an interaction (Schielzeth, 2010).

## 3 RESULTS

### 3.1 Horn growth trajectories

**Figure 3:**
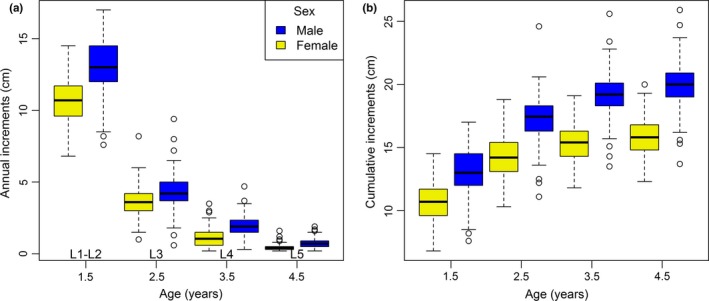
Box plots showing (a) annual increments and (b) cumulative increments of horn growth of male and female Pyrenean chamois. The first two increments (L1–L2) were formed between birth and 1.5 years, and increment 3 (L3) grew between 1.5 and 2.5 years, etc

**Figure 4:**
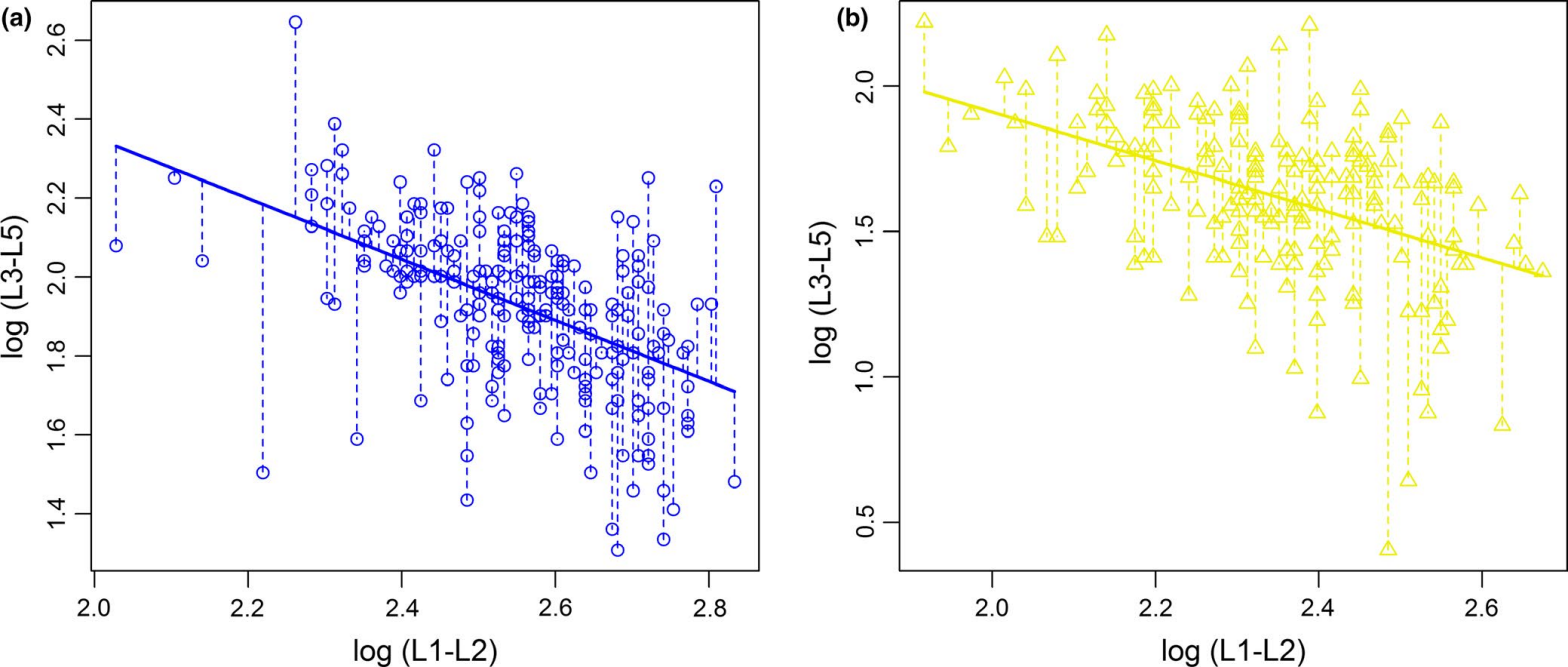
Relationship between the first two increments (L1–L2) and the three subsequent increments (from L3 to L5) on a log‐scale in (a) male and (b) female Pyrenean chamois. Residuals (marked by the dashed lines) correspond to relative late horn growth

Males had consistently longer increments than females (Figure 3a), but there was an interaction between sex and age on annual horn growth (*F*_3, 1293_ = 90.98, *p* < .001). Between the age of 1.5 and 4.5, horn increased by an average of 5.2 cm (49%) in females and 6.9 cm (53%) in males (Figure 3b). A negative relationship between the first two increments (L1‐L2) and the three subsequent increments was observed both in males (on a log‐scale, β ± *SE* = −0.77 ± 0.09, *p* < .001, Figure 4a) and females (on a log‐scale, *β* = −0.84 ± 0.13, *p* < .001, Figure 4b), and the difference between the slopes for the two sexes was not significantly different (*β* = 0.06 ± 0.15, *p* = .68). Despite this, horn length (L1–L5) was positively correlated with L1‐L2 in males (on a log‐scale, *β* = 0.36 ± 0.03, *p* < .001) and females (on a log‐scale, *β* = 0.39 ± 0.04, *p* < .001).

### 3.2 Sex‐specific relationships between horn length and survival metrics

**Figure 5:**
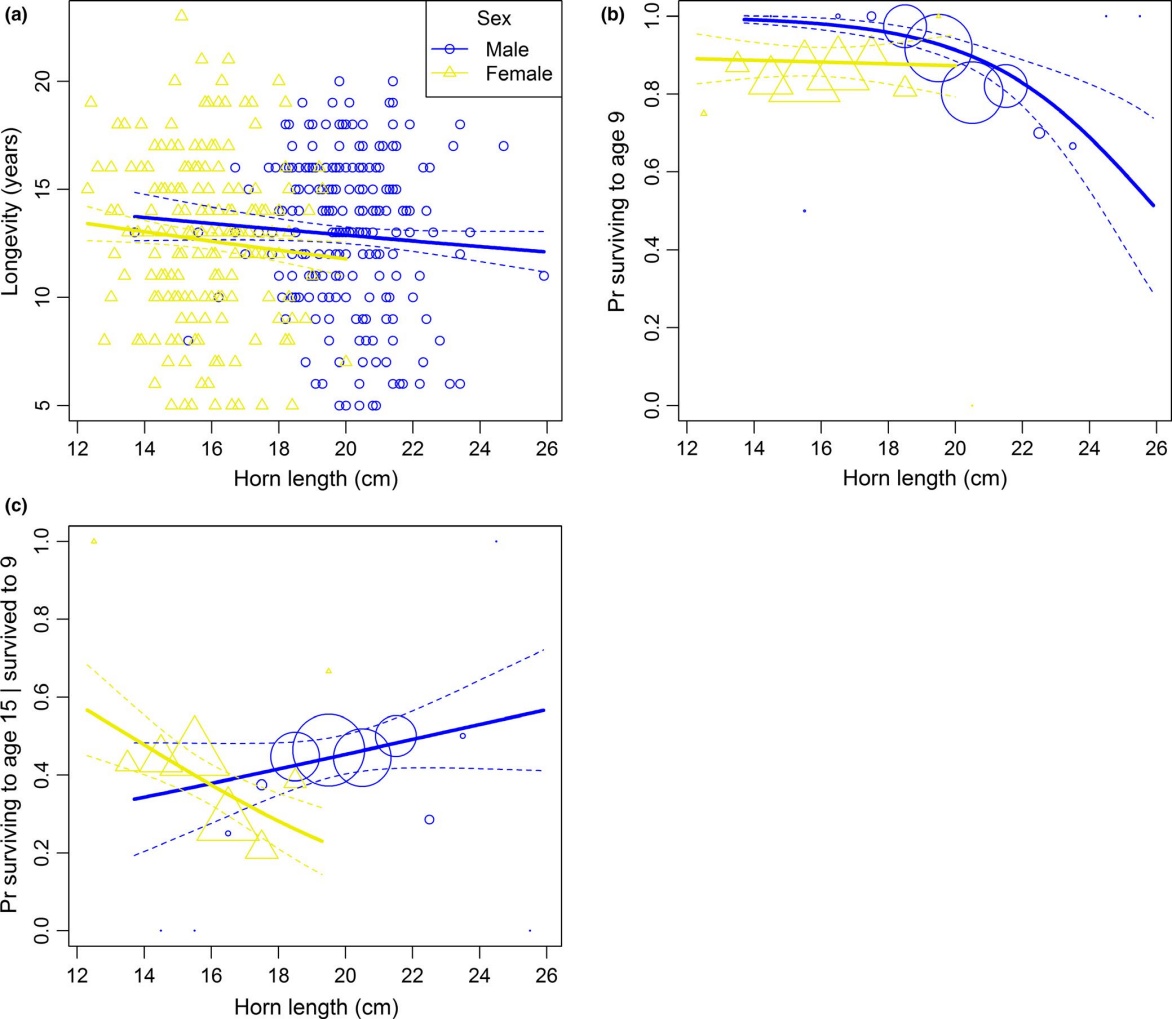
Effects of horn length on (a) longevity, (b) probability of reaching 9 years of age, and (c) probability of reaching 15 years (conditional on having survived to age 9) for males and females Pyrenean chamois. Dashed lines represent standard errors around model's predictions. In panel a, points indicate raw data. In panels b and c, circles (of different size according to the sample size) indicate average survival probabilities for each class of 1‐cm horn length

In males, horn length was not associated with longevity (*β* = −0.027 ± 0.031, *p* = .39, Figure 5a) or probability of reaching age 15 | survived to age 9 (*β* = 0.20 ± 0.25, *p* = .44, Figure 5c). However, growing long horns strongly reduced the probability of reaching 9 years of age (*β* = −1.01 ± 0.40, *p* = .01, Figure 5b). In females, there was no evidence that horn length was an important predictor of longevity (*β* = −0.044 ± 0.037, *p* = .23, Figure 5a) or probability of reaching 9 years of age (*β* = −0.058 ± 0.40, *p* = .88, Figure 5b). Females with long horns that survived to age 9 only tended to have a lower probability of reaching 15 years of age (*β* = −0.54 ± 0.31, *p* = .08, Figure 5c).

### 3.3 Sex differences in the relationships between horn length and survival metrics

Formal comparison of the sex‐specific estimates suggests that the relationships between horn length and probability of reaching 9 and 15 years of age tended to differ between sexes (9 years: *β* = −0.95 ± 0.56, *p* = .09; 15 years: *β* = 0.73 ± 0.40, *p* = .07). There was no trend for sex differences in the relationship between horn length and longevity (*β* = 0.017 ± 0.048, *p* = .72).

### 3.4 Sex‐specific relationships between early/late horn growth and survival metrics

In both sexes, we found no interaction between L1–L2 and relative late horn growth on longevity, and probabilities of reaching 9 and 15 years of age (Table 1). In males, faster growth both early and late in the growing period tended to be associated with a lower probability of reaching 9 years of age (Table 1).

**Table 1:** Effects (±standard error) of the first two increments (L1–2), relative late horn growth (residuals of the regression between log‐transformed L1–L2 and log‐transformed L3–L5, Figure 4), and their interaction on longevity and probability of reaching 9 or 15 years of age in male and female adult Pyrenean chamois

	L1−2		Late horn growth		L1–L2 × Late horn growth	
	β ± *SE*	*p*	*β* ± *SE*	*p*	*β* ± *SE*	*p*
Male						
Longevity	−0.01 ± 0.02	.51	−0.02 ± 0.02	.29	−0.02 ± 0.02	.30
Pr (surviving to age 9)	−0.44 ± 0.24	.06	−0.43 ± 0.24	.08	−0.04 ± 0.20	.83
Pr (surviving to age 15 | survived to 9)	0.12 ± 0.15	.41	0.08 ± 0.15	.56	−0.04 ± 0.12	.76
Female						
Longevity	−0.03 ± 0.02	.15	−0.01 ± 0.02	.54	0.01 ± 0.02	.61
Pr (surviving to age 9)	−0.23 ± 0.21	.28	0.07 ± 0.22	.74	0.07 ± 0.22	.77
Pr (surviving to age 15 | survived to 9)	−0.13 ± 0.19	.50	−0.32 ± 0.20	.11	0.25 ± 0.21	.23

## 4 DISCUSSION

Using a large and representative sample of an unhunted population of Pyrenean chamois (see Section 2), we found that males and females of this weakly dimorphic species were able to grow long horns without any apparent costs in terms of longevity. However, there was evidence of a negative relationship between horn length and probability of reaching 9 years of age in males. These results, which may seem contradictory at first glance, show that a set of survival metrics can be needed for reliable assessment of the costs and benefits of particular evolutionary tactics.

Our study provides rare evidence that large horns are associated with decreased survival. Trade‐offs play a central role in evolutionary biology but remain difficult to document (Roff & Fairbairn, 2007). A meta‐analysis based on numerous studies and species of birds, insects, and fish has shown that males with larger ornaments or weapons, larger body size, or higher rates of courtship generally showed greater survival or longevity (Jennions, Møller, & Petrie, 2001). Such positive co‐covariations can arise when the relative variation in resource acquisition exceeds the relative variation in resource allocation among individuals (van Noordwijk & de Jong, 1986). In addition, trade‐offs may only be evident under poor environmental conditions (Cohen et al., 2019). In large herbivores, poor environmental conditions can originate from multiple and often inter‐related factors such as high density, harsh weather, and low food availability. Density is particularly high in our population of chamois that has been at or close to carrying capacity since the 1980s. A recent study found no significant effect of early horn growth (L1–L2) on longevity in a protected population of Alpine chamois (Corlatti et al., 2017), possibly because population density relative to resource levels was not high enough. Our results also show that longevity does not necessarily capture the mortality pattern during early adulthood. The lack of an overall effect on longevity is a consequence of the divergent responses of survival with age. With an increase in horn length, a lower proportion of males survived to 9 years whereas among survivors the probability of reaching 15 years slightly increased, although not significantly so. Similarly, in the weakly dimorphic roe deer, a strong allocation to antler growth for a given mass decreased the probability of surviving beyond 6 years of age in males, while no relationship occurred for longevity (Lemaître et al., 2018). Together with this last study, our results support the idea that a negative relationship between weapon size and survival can arise in species where sexual selection is relatively weak. By contrast, early horn growth is generally not related to age‐specific survival in highly sexually dimorphic ungulates (Bergeron et al., 2008; Bonenfant et al., 2009; Toïgo et al., 2013). The capacity to detect survival cost of growing long horns may depend more on environmental conditions experienced by the population than on intensity of sexual selection.

Our study is based on correlational evidence, and correlation does not imply causation. In males of large sexually dimorphic herbivores, horns can be energetically costly to produce and carry. For instance, in bighorn sheep, horns can represent more than 15% of a ram's body mass (Blood, Flook, & Wishart, 1970) and are a major source of heat loss during winter (Picard, Thomas, Festa‐Bianchet, & Lanthier, 1994). However, both male and female chamois carry small horns that seem unlikely to have a direct effect on survival. In line with this view, survival of individuals whose horns grew faster than “normal” after a poor early horn growth was similar to that observed in other individuals. Indeed, there was no interaction between early horn growth and relative late horn growth on survival. The causal mechanisms underlying the negative correlation between horn length and probability of reaching 9 years of age in males may take several forms but are not yet understood. Young males with longer horns may suffer cost of increased early reproductive effort. Male chamois become reproductively active at about 4.5 years of age (Garel et al., 2009). In a population of Alpine chamois, the youngest successful male was 6 years at the time of conception, although the evidence should be treated with some caution given the small sample size (Corlatti et al., 2015). Territorial males have a greater reproductive success than nonterritorial ones (Corlatti et al., 2015). During the rut, territorial males defend small clustered territories by excluding rival males and defending estrous females (Von Hardenberg, Bassano, Peracino, & Lovari, 2000). During combat, male chamois generally gore the body regions with greater risk of lethal injury such as the throat or abdomen (Locati & Lovari, 1990). A study (Corlatti et al., 2012) found no evidence that territorial males have larger horns than nonterritorial males, but this may reflect a lack of statistical power given the small sample size (*n* = 19). Fighting experience is important in a large range of taxa (Hsu, Earley, & Wolf, 2006), and this may be the case in chamois because agility and strength are thought be important factors determining fight outcome (Rughetti & Festa‐Bianchet, 2010). Male chamois with large horns may engage in competition for mating at an early age with higher risk of getting hurt due to their lack of experience. In addition to increased risk injury, male chamois during early adulthood may be exposed to additional costs of mating: an earlier depletion of their body fat reserves and a higher parasite burden (Schaschl et al., 2012). These hypotheses remain highly speculative in the absence of relevant data on age‐specific male mating effort or reproductive success in chamois. Another possibility is that males carrying large horns may suffer greater costs of attaining large body size, but adult horn length was mostly independent of body mass in Alpine chamois (Rughetti & Festa‐Bianchet, 2010).

We found no evidence for survival costs in females, apart from a tendency for those reaching the age of 9 and with long horns to have a lower probability of reaching 15 years of age. A similar tendency is found among older Alpine female chamois (Bleu et al., 2014). Ornaments and weapons can influence access to food resources via social interactions (Tobias, Montgomerie, & Lyon, 2012). For instance, in Soay sheep, horned females are more likely to initiate and win aggressive interactions than hornless ones (Robinson & Kruuk, 2007). In chamois, female dominance rank was correlated with age, body mass, and horn length, although that mass seems be the most important determinant (Locati & Lovari, 1991). The advantage in competition over resources may translate into reproductive benefits. Female Alpine chamois with strong early horn growth attained primiparity early and horn growth was positively correlated with reproduction during senescence stage (Rughetti & Festa‐Bianchet, 2011a). Large‐horned females may thus allocate more resources to reproduction, but at the cost of survival at old ages. Although recent studies in female chamois show no clear evidence of reproductive costs in any age class (Morin, Rughetti, Rioux‐Paquette, & Festa‐Bianchet, 2016; Tettamanti, Grignolio, Filli, Apollonio, & Bize, 2015), such costs may only be evident in populations experiencing poor environmental conditions.

Our results are highly relevant to management and conservation of large herbivores. By removing large‐horned males before they obtain high reproductive success, trophy hunters induced a rapid evolution of reduced horn growth in bighorn sheep (Pigeon, Festa‐Bianchet, Coltman, & Pelletier, 2016). Although it is extremely difficult to obtain data allowing tests of potential evolutionary changes of weapon size in response to selective harvesting, decreases in horn size consistent with evolutionary consequences of trophy hunting have been reported for five species of ungulates (Festa‐Bianchet & Mysterud, 2018). In chamois, if trophy hunting partly reflects natural mortality, this species would be less susceptible to evolutionary consequences of selective harvesting. Temporal declines in both horn length and body mass reported in several populations of chamois (Corlatti et al., 2017; Mason, Willis, Chirichella, Apollonio, & Stephens, 2014; Rughetti & Festa‐Bianchet, 2012) appear to be caused by climate change, rather than selective hunting.

## CONFLICT OF INTEREST

None declared.

## AUTHORS’ CONTRIBUTIONS

M.D., A.L., and C.B. designed the study. J.P.C. collected the data. M.D. analyzed the data and wrote the manuscript, with contributions from all authors.

## Data Availability

The data supporting the analyses are available from the Dryad Digital Repository. https://doi.org/10.5061/dryad.w3r2280m4.
